# The effects of traditional mind-body exercises on cognitive function in neurodegenerative diseases or prodromal cognitive decline: a meta-analysis

**DOI:** 10.3389/fpubh.2026.1735606

**Published:** 2026-02-11

**Authors:** Xin Liu, Cai Cui, Jiacan Lv, Yinhang Zhang

**Affiliations:** 1 College of Physical Education, Yangzhou University, Yangzhou, China; 2 Arts and Physical Education Department, Henan Technical College of Construction, Zhengzhou, China; 3 Department of Social Sports, Henan Sport University, Zhengzhou, China

**Keywords:** attention, cognitive function, executive function, language, memory, neurodegenerative diseases, traditional mind-body exercises

## Abstract

**Objective:**

The meta-analysis aimed to systematically assess the influence of traditional mind–body exercise (TMBE), including taichi, baduanjin, wuqinxi, and yoga, on cognitive functioning and related cognitive decline for patients with neurodegenerative diseases (NDDs) or Prodromal Cognitive Decline.

**Methods:**

Randomized controlled trials (RCTs) were published until October 19, 2025, as determined by searching PubMed, Cochrane Library, Web of Science, and Embase. The study population included adults with AD and PD, including those at prodromal stages, such as MCI and SCD. The interventions were a TMBE group and a control group. The primary outcome was overall cognitive functioning score, which was measured using the MMSE or MoCA, and secondary outcomes included executive function, memory, attention and language. Data were analyzed using random effects models and quality assessed with the Cochrane Risk of Bias Tool.

**Results:**

Twenty-one RCTs were included, with the total sample size varying according to outcome metrics. TMBE showed significant improvement in overall cognitive functioning (MMSE: MD = 0.65, 95% CI: 0.20 to 1.09, *p* = 0.004; MoCA: MD = 0.87, 95% CI: 0.46 to 1.29, *p* = 0.001). Significant differences were seen in executive function (e.g., digit reversal: MD = 0.24, 95% CI: 0.05 to 0.44, *p* = 0.013; TMT-B: MD = −1.18, 95% CI: −1.70 to −0.67, *p* < 0.001), verbal fluency (MD = 0.36, 95% CI: 0.14–0.57, *p* = 0.001), and significant benefits were also observed in specific subgroups with respect to long-term delayed recall (e.g., mild dementia: MD = 1.35, 95% CI: 0.81–1.88, *p* < 0.001). Attention improvement effects were generally positive but varied by assessment tool. Moderate to high heterogeneity existed for some of the outcome indicators, but this tended to be resolved after sensitivity analyses. The degree of publication bias was low.

**Conclusion:**

TMBE has demonstrated the ability to contribute to improvement of overall and specific cognitive functions in individuals diagnosed with NDDs or Prodromal Cognitive Decline. These trainings offer a promising non-pharmacological intervention strategy that is safe, reliable, and multi-targeted to improve cognitive impairment in this population.

**Systematic review registration:**

PROSPERO – International prospective register of systematic reviews Unique identifier: CRD420251106629, https://www.crd.york.ac.uk/prospero/display_record.php?ID=CRD420251106629.

## 1 Introduction

Neurological disorders encompass a broad spectrum of conditions affecting the central and peripheral nervous systems, spanning from common diseases like AD and PD to rarer forms such as HD (1, 2). Global Burden of Disease studies have shown that these diseases account for a significant proportion of the total disease burden and have become one of the major contributors to the loss of disability-adjusted life years (3). Their social and economic impact even surpasses that of many other disease categories (3). Globally, the demographic landscape is increasingly marked by population aging, which is accompanied by an increasing incidence of neurodegenerative diseases and a heavy burden of disease (4). These disorders are often accompanied by impairments in cognitive, motor, or sensory functions, which severely compromise both the physical health and quality of life of patients (5, 6).

Cognitive function involves advanced mental processes by which the brain receives, processes, stores, and utilizes external information. It covers a number of areas such as memory, executive function, attention, language and visuospatial abilities (7–9). The core pathological mechanisms of NDDs—including abnormal protein aggregation (10, 11), neuroinflammatory responses (12, 13), and synaptic structural and functional impairments (14), directly leading to progressive decline in cognitive function. Notably, although feature of PD and HD are primarily motor signs including resting tremor and choreiform movements, their effects on cognitive function are equally significant (15–17). These cognitive impairments often emerge early in the disease course yet are frequently overlooked or underestimated due to their subtle presentation. Although available medications provide some relief from some motor or cognitive symptoms, they usually do not stop disease progression or restore function. Moreover, prolonged pharmacotherapy carries the risk of adverse events (17, 18). Therefore, it is necessary to explore and evaluate non-pharmacological intervention strategies, in ensuring their safety and efficacy.

For the past few years, exercise intervention has been taken on the role of a promising non-pharmacological strategy to effectively enhance motor function and cognition within patients with NDDs (19–21). Among varieties of exercise, TMBE, represented by yoga, taichi, baduanjin, and wuqinxi, are gaining attention. As early as the Ming and Qing dynasties in China, the scholar-official and merchant classes played a central role in the advocacy and practice of traditional mind–body exercises, fostering an environment in which such practices became deeply embedded within daily life and cultural values. This type of exercise is known for its gentle movements, focuses on mind–body coordination, and incorporates breathing regulation techniques (22–24). These characteristics make them particularly suitable for long-term practice among older adults and neurologically impaired populations (25). Such exercises combine elements of moderate-intensity aerobic activity, balance training, attentional focus, and controlled breathing (26, 27). Through mechanisms such as the modulation of neurotrophic factors (28), enhancement of cerebral perfusion (29), reinforcement of neural connections (30), and attenuation of neuroinflammation (31), they may not only improve physical fitness but also support cognitive health. Although several empirical studies have shown that these practices help delay cognitive decline and enhance executive function and memory (25), the evidence remains inconclusive. In addition, existing research results still need to be strengthened in accordance with systematic analysis and quantitative assessment.

In view of this, our study was aimed at critically evaluating effectiveness of TMBE on cognitive function in older adults with neurodegenerative disease spectrum disorders and preclinical cognitive impairment, which could advance cognitive ability for sufferers with NDDs relying on our study. Findings of this research support firmly for the application and dissemination of interventions, and are expected to drive a shift in clinical practice and health policy making toward higher standards of evidence. It should be noted that significant cognitive impairment may also occur in the early stages of these diseases, such as during the MCI phase.

## 2 Materials and methods

### 2.1 Design and eligibility criteria

This study strictly followed the Preferred Reporting Items for Systematic Evaluation and Meta-Analysis (PRISMA) guidelines. The study was prospectively registered with the PROSPERO platform (registration number: CRD420251106629).

Literature screening and inclusion were done independently by two researchers. Discrepancies were addressed through discussion, with the option to escalate to an independent arbitrator for final resolution.

The inclusion criteria were shown below:

1. Category of research: RCTs from peer-reviewed, English journals;
2. Participants: This study included adult patients across the entire spectrum of cognitive decline, encompassing those with diagnosed neurodegenerative diseases—such as AD and PD, as well as those at high risk or in prodromal stages, such as MCI and SCD. This inclusive design aims to comprehensively evaluate the potential of TMBE, both to improve cognition in patients with diagnosed neurodegenerative diseases and to prevent or delay disease progression in its early stages.
3. Intervention: The main intervention in the experimental group was the TMBE program, which included Tai Chi, wuqinxi, baduanjin, and Yoga;
4. Control: The control group could receive passive control, active control, or other non-exercise interventions;
5. Outcome measures: Studies must report on indicators of cognitive function, covering cognition, memory, attention, executive function, and language skills.

The exclusion criteria were:

1. Studies with no RCTs, incomplete data, or where key effect size data could not be obtained;
2. Interventions involving single acute exercise sessions, or where mind–body exercises were combined with other interventions preventing independent evaluation of their effects;
3. Studies involving participants with comorbid neurological, metabolic diseases, or psychiatric disorders that could significantly affect cognition;
4. Non-English publications or without full-text availability.

### 2.2 Literature search and selection

To ensure comprehensive collection of relevant studies, we systematically searched multiple Chinese and English electronic databases. English databases included Embase, PubMed, the Cochrane Library, and Web of Science. Articles published from the inception of each database through July 30, 2025, were included in English language. An updated search was performed on October 19, 2025. The full search strategy is provided in Supplementary Table S1. Literature screening was done independently by two researchers. Duplicate records were initially eliminated through the reference management software, and the remaining records were subsequently screened title by title, abstract by abstract, and full text by full text. All disagreements were settled via team consultation or, failing which, by recourse to an independent adjudicator.

### 2.3 Data extraction

Data extraction was performed in line with the Cochrane Handbook. The process was completed independently by two researchers utilizing a pre-planned standardized data extraction sheet in Microsoft Excel. Disagreements were worked out by jointly reviewing the original article or third-party adjudication. The extracted information mainly includes: basic study characteristics, participant characteristics, intervention details, including specific type of TMBE, session duration, frequency, total intervention period, and post-intervention mean, standard deviations, and sample sizes for cognitive domain tests. Overall cognitive domains mainly include executive function, language ability, attention and memory. In this study, we mainly extracted the mean and standard deviation of each group post-intervention to analyze the intervention effect of TMBE for cognitive function.

### 2.4 Quality assessment

**Figure 1:**
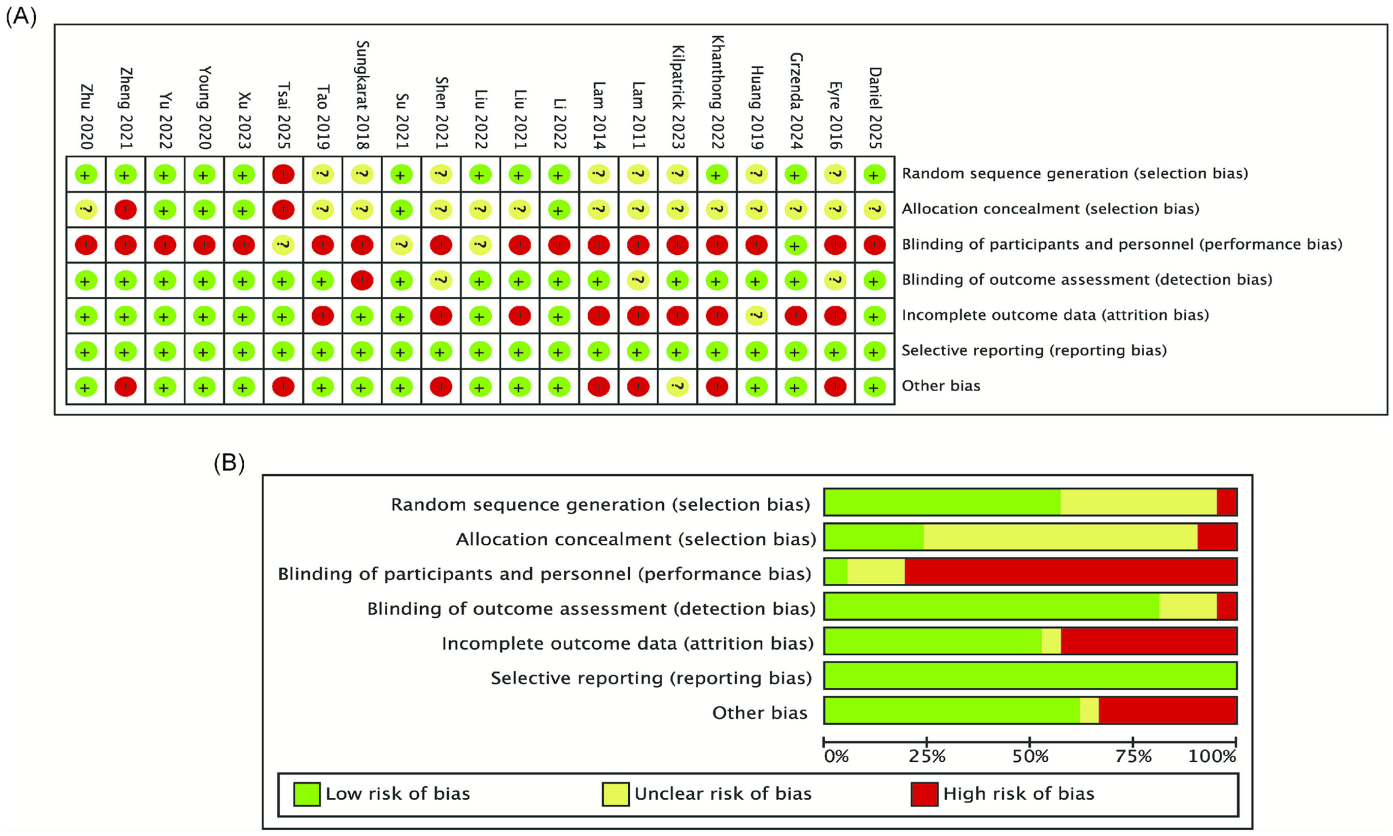
Risk of bias assessment for included studies: **(A)** risk of bias summary; **(B)** risk of bias graph.

The methodological quality including RCTs was evaluated by the Cochrane Risk of Bias Assessment Tool (RoB 2). This tool assesses seven fields (Figure 1). The work was completed independently by two researchers. Each field was assessed as ‘low risk of bias,’ ‘unclear risk of bias,’ or ‘high risk of bias’ according to the RoB 2 criteria. If the two evaluators did not agree, consensus was reached through joint deliberation or arbitration by a third researcher. The overall risk of bias judgment for each study was assigned according to its comprehensive domain scores. Detailed assessment results are shown in Figure 1.

### 2.5 Statistical analysis

All meta-analysis were conducted by Stata 18.0 and Reviewer manager 5.4. Effect sizes were combined and analyzed using a random effects model to better accommodate heterogeneity among studies regarding exercise type and population characteristics. To investigate potential sources of heterogeneity, Sufficient studies were available (*k* ≥ 10), meta-regression analyses were performed to explore the influence of continuous variables on effect sizes, using the metareg command in Stata with REML estimation and Knapp-Hartung adjustment. Subgroup analyses were conducted for categorical variables such as intervention type and participant diagnosis.

For continuous outcome measures related to cognitive function, when all studies employed the same assessment tools and units by using MMSE or MoCA scale scores, we pooled the mean differences. When studies assessed the same cognitive construct using different tools such as employing multiple distinct memory tests, we pooled the standardized mean differences. Both are reported with their 95% confidence intervals (CI). Heterogeneity was quantified using the *I*^2^ statistic and the Cochran’s *Q* test, with *I*^2^ > 50% or a *Q*-test *p*-value < 0.10 indicating substantial heterogeneity.

Sensitivity analyses were conducted by sequentially the one-by-one exclusion method to assess whether results were unduly influenced by specific studies. If heterogeneity significantly decreased or the pooled effect size changed importantly after removal, it suggested that study might be a major source of heterogeneity. The robustness of the findings was evaluated through iterative exclusion of individual studies and reassessment of heterogeneity in the pooled estimates. To assess potential publication bias, Egger’s test was used and assessed by visual inspection of funnel plots. If data were incompletely reported in the original studies, such as means and standard deviations, corresponding authors were contacted using e-mail to obtain information for the analysis. If it cannot be obtained from the authors, we plan to calculate and convert the data using standard error, confidence intervals, or *p*-values provided in the original articles, following the guidelines in the Cochrane Handbook.

## 3 Results

### 3.1 Search results

**Figure 2:**
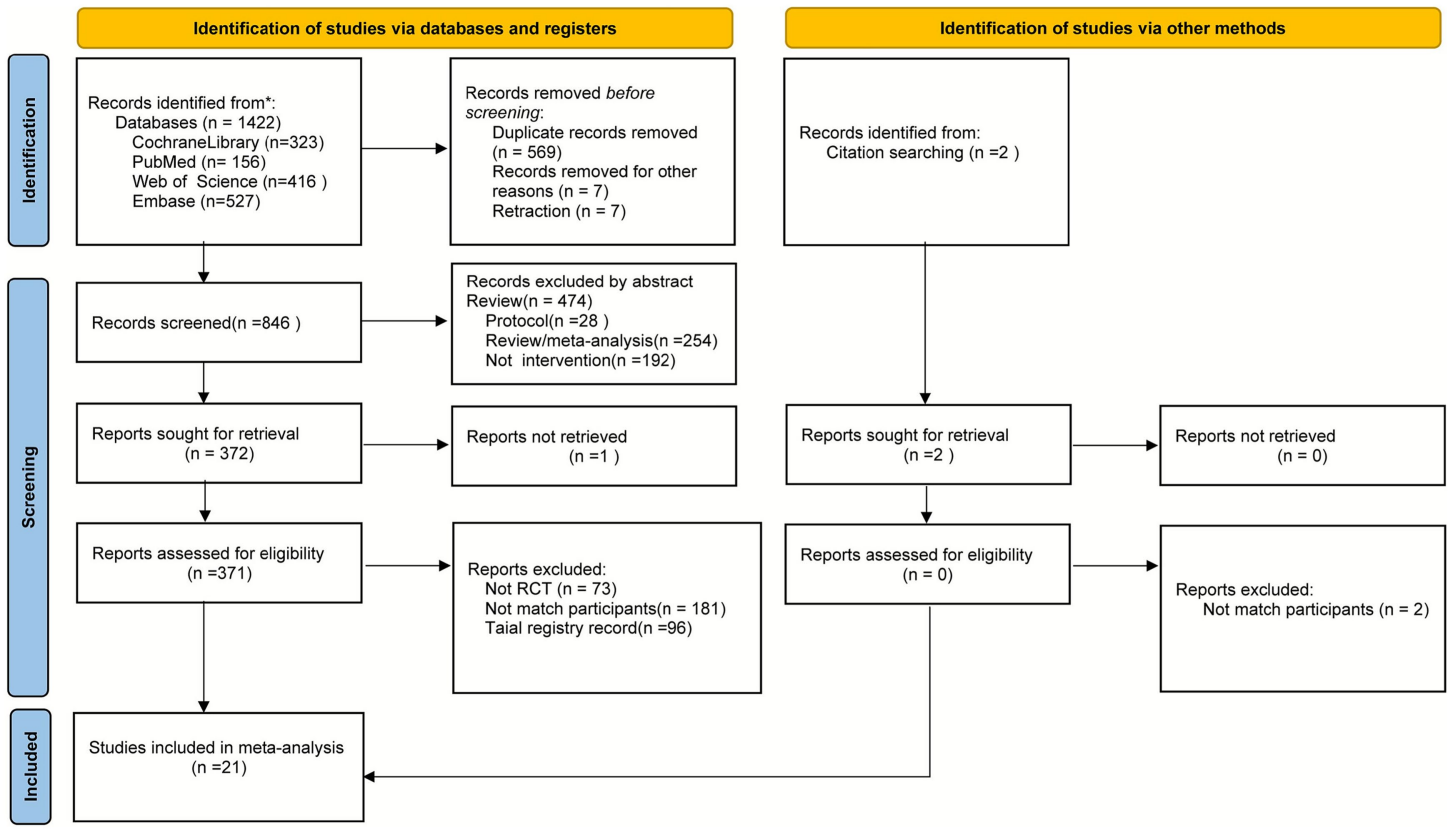
PRISMA flow chart of the literature search and selection procedure. *n* = number.

A systematic literature search initially identified 1,422 potentially relevant records. Before screening, 569 duplicate records and 7 retracted records were removed. Subsequently, titles and abstracts of 846 records were screened, leading to the exclusion of 474 irrelevant records, which included 28 study protocols, 254 reviews/meta-analyses, and 192 non-interventional studies. We attempted to retrieve complete text of the remaining 372 records, of which 1 was unavailable. The full text of 371 articles underwent rigorous assessment for compliance with the study. The main reasons for exclusion include: ineligible study design (e.g., non-RCTs, trial registry entries without resultant publications, total *n* = 169), and ineligible population (*n* = 181). Furthermore, a citation search turned up 2 more records, but both were excluded after assessment due to ineligible population. Ultimately, the meta-analysis included 21 studies. The research selection process was shown in the PRISMA flowchart (Figure 2).

### 3.2 Characteristics of included studies

Details of the 21 studies (32–52) included in our study are listed in Table 1, which were published across the years 2011 to 2025. All studies had varying sample sizes (22–389), but all covered both male and female populations. The participants included individuals with MCI (*K* = 12), PD (*K* = 2), AD (*K* = 1), and other types of cognitive impairment, including a-MCI (*K* = 1), MD (*K* = 2), MCI&SMC (*K* = 1), SMC (*K* = 1), and MCI&SCD (*K* = 1).

**Table 1:** Characteristics of the included studies.

Authors	Design	Population	Intervention (n)	Intervention schedule	Cognitive domains and assessments	Key cognitive findings
Lam et al. (32), Hong Kong China	RCT (*n* = 329)	Older adults with MCI, aged ≥60 years	Tai Chi (*n* = 135) Control (*n* = 194)	3x/week, 30 min, 3 months	Global: CDR-SB, MMSE; Language: CVFT; Memory: Delayed Recall; Attention: Visual Span	Both groups improved in MMSE, ADAS-Cog Delayed Recall, CVFT at 5 months; TC group showed greater improvements in CDR-SB and Visual Span
Lam et al. (33), Hong Kong China	RCT (*n* = 261)	Older adults with MCI, aged ≥65 years	Tai Chi (*n* = 92) Control (*n* = 169)	3x/week, 30 min, 12 months	Global Cognition: CDR, CDR-SB MMSE, ADAS-Cog Memory: Delayed Recall, Digit Span, Visual Span Language: Category Verbal Fluency Executive Function: TMT B	TC group had lower dementia conversion rate, better CDR-SB preservation, and greater improvement in delayed recall
Lam et al. (34), Hong Kong, China	RCT (*n* = 389)	older adults with MCI, aged ≥65 years	Tai Chi (*n* = 171) Control (*n* = 218)	3x/week, 30 min, 12 months	Executive: TMT-B; Global: CDR-SB, MMSE; Language: CVFT; Memory: Delayed Recall, Digit Span	No significant between-group differences in MMSE; improvements in Digit Span, Delayed Recall, CVFT, and CDR were observed in both groups
Eyre et al. (35), UCLA, USA	RCT (*n* = 25)	Older adults with a-MCI, aged ≥ 55 years	Yoga (*n* = 14) Control (*n* = 11)	1time/week, 50 min, 12 months	Memory: Verbal Memory, Visuospatial Memory	Yoga group showed significant improvement in visuospatial memory
Sungkarat et al. (36), Chiang Mai, Thailand	RCT (*n* = 66)	Older adults with amnestic multi-domain MD aged 67.9 years	Tai Chi (*n* = 33) Control (*n* = 33)	3x/week, 50 min, 15 weeks total	Memory: Verbal Memory, Visuospatial Memory	TC group showed significantly greater improvement in delayed recall
Huang et al. (37), Beijing, China	RCT (*n* = 74)	Older adults with MCI&SMC, aged ≥60	Tai Chi (*n* = 36) Control (n = 38)	3x/week, 20 min, 10 months	Memory: delayed recall; Executive: Digit Span backward, TMT B-A; Attention: Digit Span forward	TC group demonstrated significantly greater improvement in delayed recall and TMT B-A
Tao et al. (38), Fujian, China	RCT (*n* = 40)	Older adults with MCI, aged ≥60 years	Baduanjin (*n* = 23) Control (*n* = 23)	2x/week, 60 min, 24 weeks	Global: MoCA	Baduanjin group showed significantly increased MoCA scores
Zhu et al. (40), Hangzhou, China	RCT (*n* = 41)	Patients with mild-to-moderate PD aged 45 to 80 years	Tai Chi (*n* = 19) Control (*n* = 22)	3x/week, 40–50 min, 12 weeks	Global: MoCA	TC group showed significantly greater improvement in MoCA than control
Young (39), Hong Kong, China	RCT (*n* = 80)	Older adults with MD, aged ≥60 years	Baduanjin (*n* = 41) Control (*n* = 39)	2x/week, 60 min, 14 weeks	Memory: delayed recall; Executive: Digit Span backward, TMT B-A; Attention: Digit Span forward	Intervention group showed significantly greater improvements in DRS total score, MMSE, and memory subscores
Liu et al. (41), Fuzhou, China	RCT (*n* = 46)	adults with MCI, aged ≥60 years	Baduanjin (*n* = 23) Control (*n* = 23)	3x/week, 60 min, 24 weeks	Global: MoCA	Baduanjin group had significantly greater increase in MoCA scores than control
Shen et al. (42), Shanghai, China	RCT (*n* = 30)	PD aged between 55 and 80 years	Wuqinxi (*n* = 15) Control (*n* = 15)	2x/week, 90 min, 12 weeks	Global: MoCA; Executive: Stroop I & II	Wuqinxi group showed significant improvements in Stroop I, Stroop II, and MoCA scores after intervention
Zheng et al. (44), Fuzhou, China	RCT (*n* = 46)	Older adults with MCI, aged ≥60 years	Baduanjin (*n* = 23) Control (n = 23)	3x/week, 60 min, 24 weeks	Global: MoCA; Memory: WMS-CR	Baduanjin had significantly higher total scores on MoCA and WMS-CR
Su et al. (43), Daqing, China	RCT (*n* = 65)	Older adults aged 60 to 75 years with SMC	Baduanjin (*n* = 32) Control (*n* = 33)	5x/week, 60 min, 12 weeks	Global: MoCA; Memory: SMCQ, AVLT; Executive: TMT-B; Attention: TMT-A	Baduanjin group showed significant improvements in SMCQ, AVLT total scores, and TMT
Khanthong et al. (45), Thailand	RCT (*n* = 66)	Adults aged between 50 and 80 years with MCI	Ruesi Dadton (RD) (*n* = 34) Control (n = 32)	3x/week, 60 min, 12 weeks	Cognitive: MMSE; MoCA	RD group had higher baseline MoCA; showed improvement in oxidative stress markers post intervention
Liu et al. (46), Taipei, China	RCT (*n* = 34)	Older adults with MCI, aged ≥ 65 years	Tai Chi (*n* = 17) Control (*n* = 17)	3x/week, 50 min, 12 weeks	Global: MoCA; Executive: TMT-B, SCWT; Attention: TMT-A	TC group improved in TMT, SCWT, and one- back test compared to control
Yu et al. (47), Hong Kong, China	RCT (*n* = 22)	Adults with MCI, aged ≥50 years	Tai Chi (*n* = 10) Control (*n* = 12)	3x/week, 60 min, 24 weeks	Global: HK-MoCA; Executive: TMT-B; Attention: Digit Span forward, TMT-A; Language: Verbal Fluency	TC group showed greater improvement in cognitive flexibility (TMT B/A ratio) at 12 and 24 weeks
Kilpatrick et al. (48), Los Angeles, USA	RCT (*n* = 22)	Women aged between 61 and 65 years with AD	Kundalini yoga (*n* = 11) Control (*n* = 11)	1 time/week, 60 min, 12 weeks	Memory: MFQ	KY showed potential protective benefits for long-term episodic memory
Xu et al. (49), Fuzhou, China	RCT (*n* = 92)	Participants aged between 50 and 75 years with MCI	Tai Chi (*n* = 49) Control (*n* = 43)	3x/week, 60 min, 12 weeks	Cognitive: MoCA; Memory: MQ, AVLT; Executive: Stroop test	TC group showed greater improvement in MoCA, MQ, AVLT immediate recall, Stroop color time
Grzenda et al. (50), Los Angeles, USA	RCT (*n* = 79)	Women with MCI&SCD, aged ≥ 50 years	Kundalini yoga (*n* = 40) Control (*n* = 39)	3x/week, 60 min, 12 weeks	Memory: Delayed recall; Executive: TMT-B	At 24 weeks, KY showed significant improvement in MFQ Factor 2 versus control; demonstrated decline in delayed recall at 24 weeks
Daniel et al. (51), Hong Kong, China	RCT (*n* = 57)	Older adults with MCI, aged ≥60 years	Baduanjin (*n* = 30) Control (*n* = 27)	2x/week, 15 min, 12 weeks	Cognitive: MoCA total score and subdomains (memory, attention, language)	At the follow-up at 8 months and 6 months after the intervention, MoCA and memory decall were significantly increased
Tsai et al. (52) Taiwan, China	RCT (*n* = 24)	Patients with MCI, aged ≥65 years	Baduanjin (*n* = 12) Control (*n* = 12)	3x/week, 40–50 min, 12 weeks	Cognitive: MoCA total score	MoCA: Significant improvement at 9 weeks and 13 weeks in experimental group

n, number; x, times; RCT, randomized controlled trial; TC, Tai chi; KY, Kundalini yoga.

### 3.3 Quality of included studies

Twenty-one RCTs were included for methodological quality assessment. Across the seven bias domains, most studies were judged as low risk concerning random ‘sequence generation’, ‘blinding of participants and personnel’, and ‘completeness of outcome data’. Specifically, regarding random sequence generation, approximately half of the studies were low risk, while the others were rated as having an unclear risk, as a result of insufficient description of the randomization method. Concerning allocation concealment, most studies raised concerns due to inadequate description of the allocation concealment method. In the light of blinding implementation, for blinding of participants and personnel (performance bias), most studies were low risk, with a few being unclear; for blinding of outcome assessment (detection bias), most studies were low risk, while some were unclear. For selective reporting, the vast majority of studies were low risk, with only individual studies rated as high risk. Furthermore, all studies were rated as low risk in the ‘other bias’ domain. The qualitative aspects of the methodology in the studies included was generally acceptable, with the primary potential sources of bias related to inadequate reporting of allocation concealment and incomplete details regarding the implementation of some blinding procedures. More detailed assessment results are shown in Figure 1.

### 3.4 Primary outcome: global cognitive function

A comprehensive assessment of global cognitive function was conducted through a combination of the MMSE and MoCA. Five studies (total 814 participants, intervention/control: 342/472) assessed the intervention effect on global cognitive function. A random-effects meta-analysis of MMSE scores showed a significant advantage for the intervention group over the control group (MD = 0.65, 95% CI: 0.20 to 1.09, *p* = 0.004), but there was high heterogeneity between studies (*I*^2^ = 87.6%, *p* < 0.001). Assessment of treatment effect heterogeneity by disease type confirmed that significant improvement was preserved within the relevant subgroup (MD = 0.44, 95% CI: 0.30 to 0.58, *p* = 0.000). Among participants with MCI, MMSE scores showed a trend toward improvement but did not reach statistical significance (MD = 0.32, 95% CI: 0.17 to 0.48, *p* = 0.195), and heterogeneity remained high (*I*^2^ = 58.3%, *p* = 0.091). Notably, a substantial benefit effect was observed in the Mild Dementia (MD) population (MD = 1.76, 95% CI: 1.24 to 2.28, *p* = 0.000). This high degree of heterogeneity may stem from the diversity of the included populations, ranging from MCI to mild dementia and variations in intervention types, such as Tai Chi and Baduanjin. Subgroup analyses revealed the largest effect size and lowest heterogeneity in the mild dementia cohort, indicating that disease severity is a significant influencing factor.

**Figure 3:**
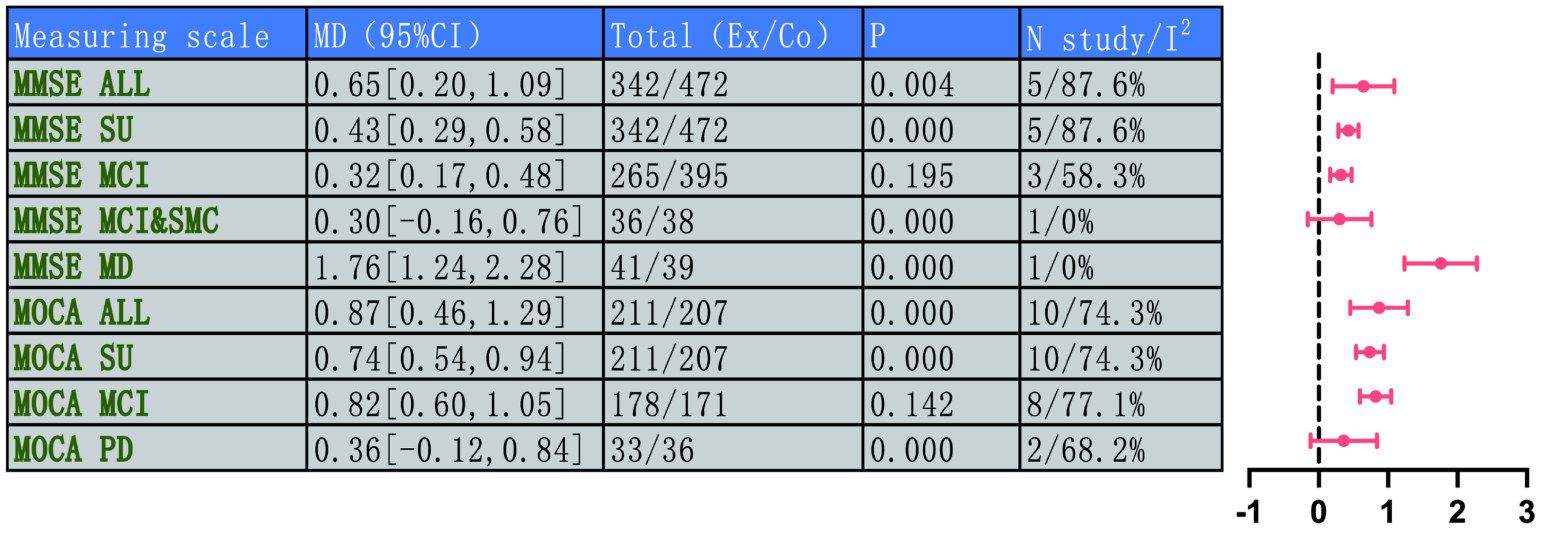
Comprehensive forest plots of overall cognitive function.

**Figure 4:**
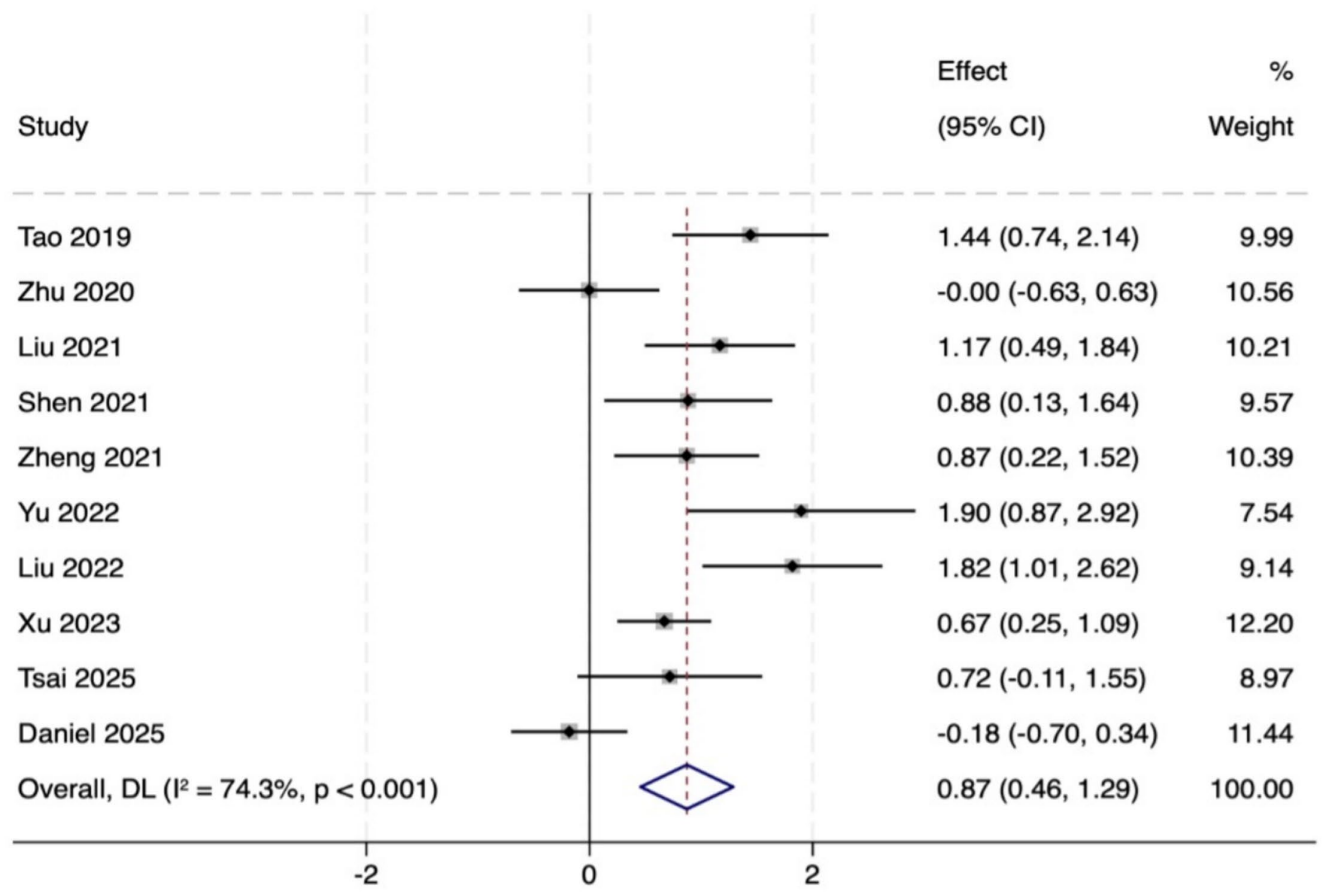
Forest map of MOCA scale.

**Figure 5:**
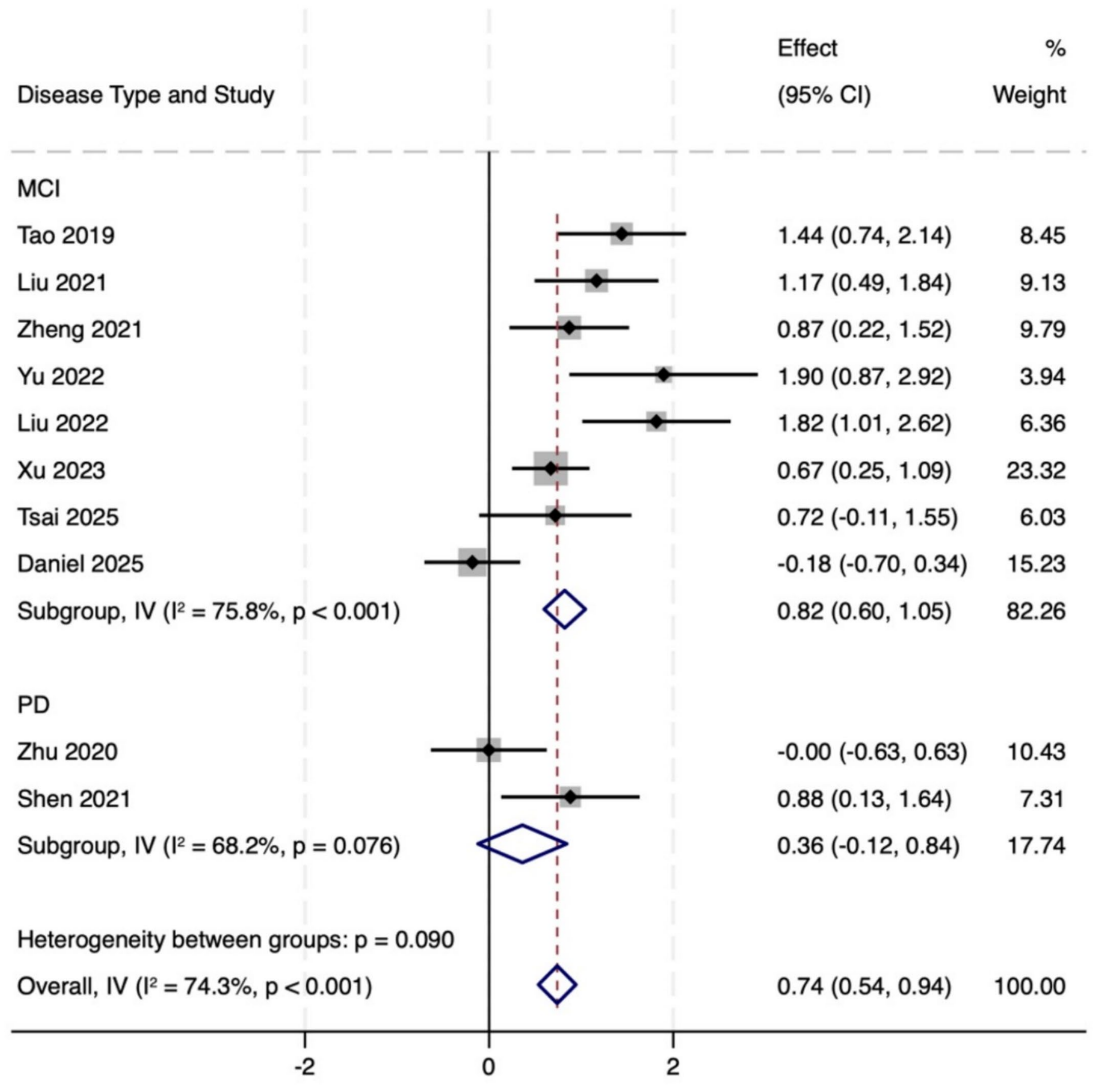
Subgroup analysis forest map of MOCA scale by participants.

**Figure 6:**
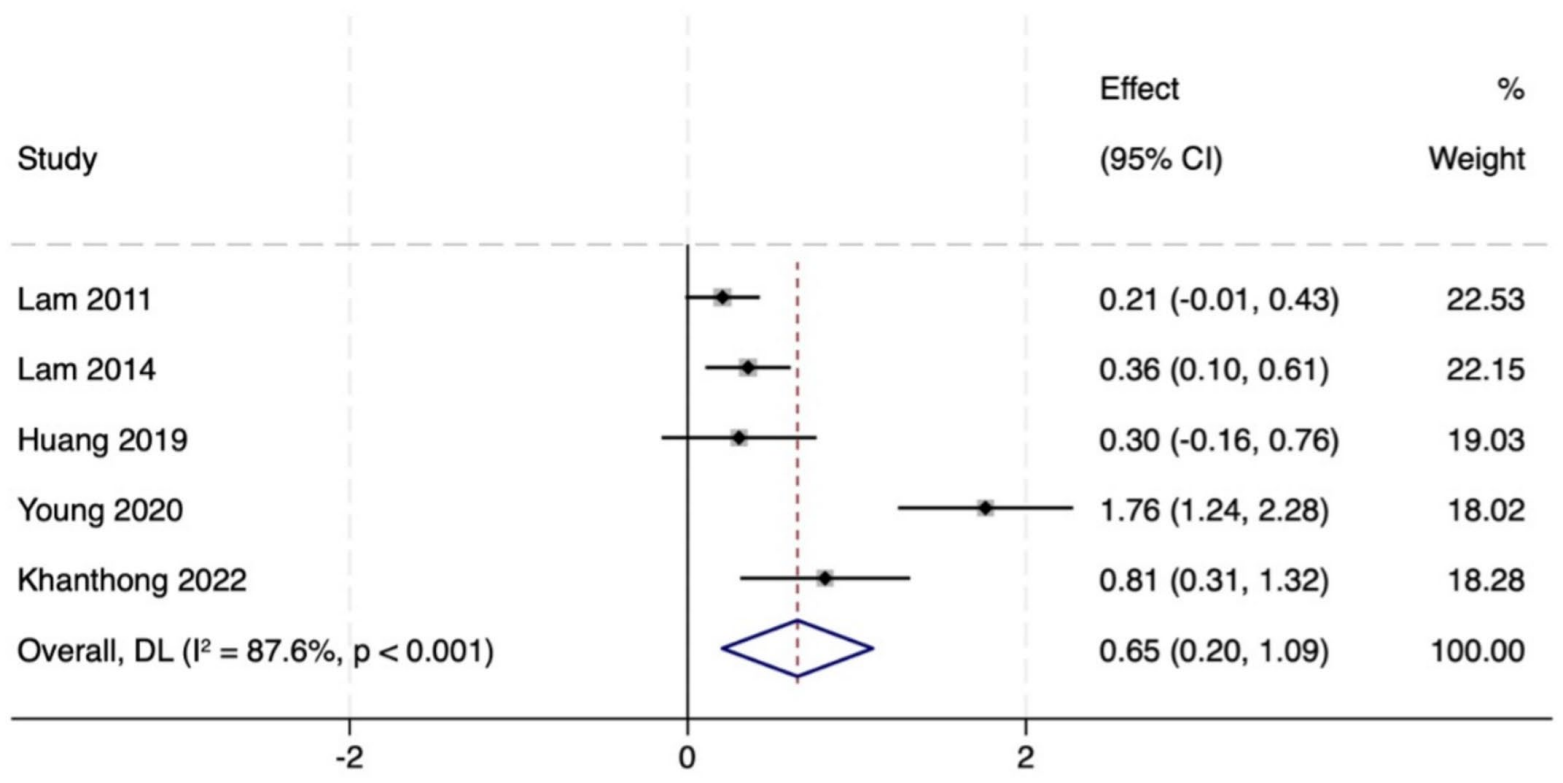
Forest map of MMSE scale.

**Figure 7:**
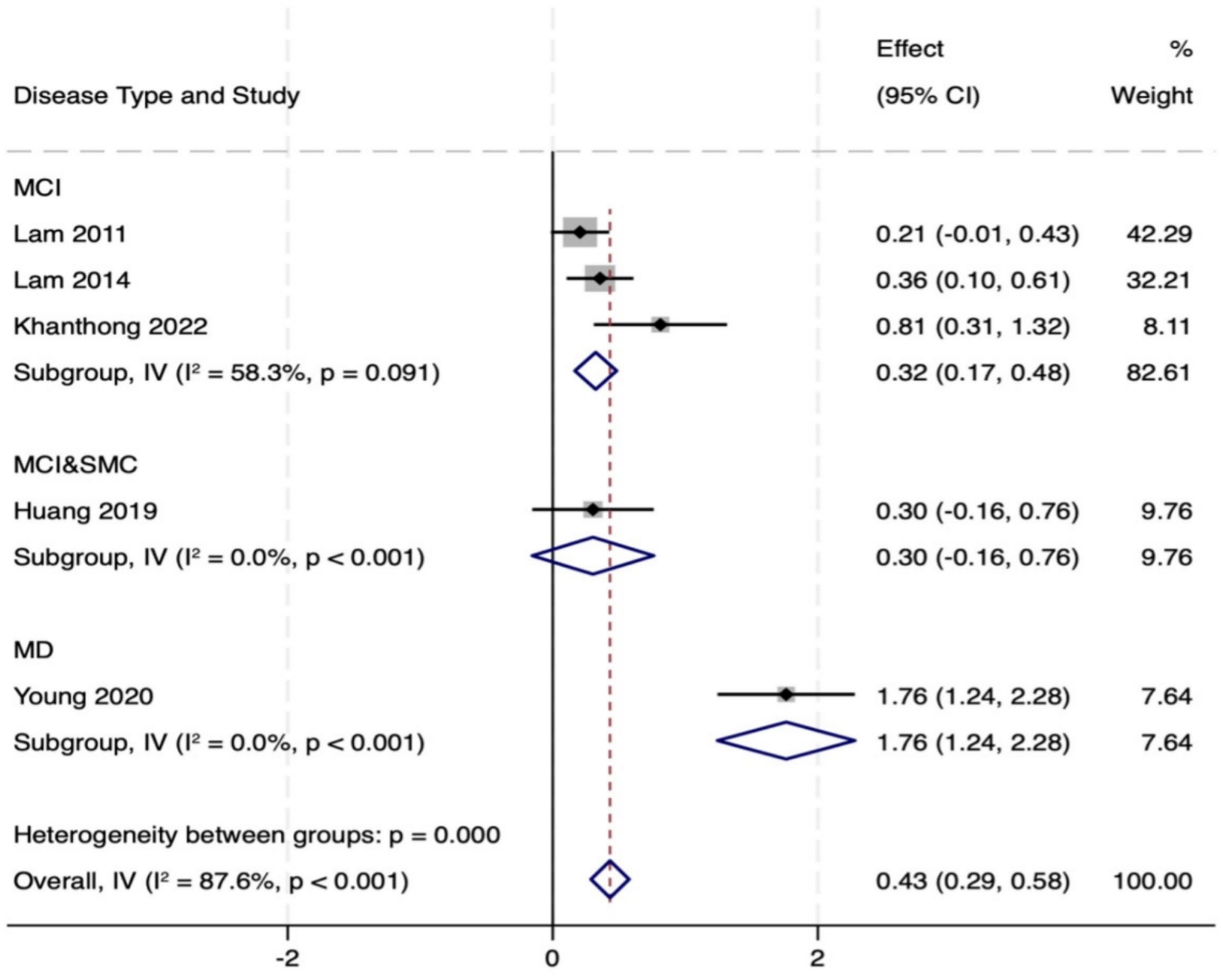
Subgroup analysis forest map of MMS scale.

Ten studies (total 419 participants, intervention/control: 211/207) used the MoCA to assess global cognitive function. Pooled analysis revealed that MoCA scores in the intervention group indicated markedly better versus control group (MD = 0.87, 95% CI: 0.46 to 1.29, *p* = 0.000), with moderate heterogeneity (*I*^2^ = 74.3%, *p* < 0.001). This subgroup analysis also showed remarkable improvement (MD = 0.74, 95% CI: 0.54 to 0.94, *p* = 0.000). In the MCI population, MoCA scores increased significantly (MD = 0.82, 95% CI: 0.60 to 1.05, *p* = 0.142), but heterogeneity was high (*I*^2^ = 77.1%). In PD patients, scores in the intervention group showed an increasing trend, however, the observed differences did not reach statistical significance (MD = 0.36, 95% CI: −0.12 to 0.84, *p* = 0.000). In summary, TMBE conferred substantial benefits on global cognitive function, the MD subgroup exhibited the most pronounced response. Detailed results are shown in Figures 3–7.

To further explore potential sources of MoCA score heterogeneity, we conducted meta-regression analyses for two continuous variables, the mean age of participants and total intervention duration (Supplementary Figure S1). These variables were selected based on their theoretical association with cognitive outcomes and data availability across studies.

Meta-regression analysis for mean age revealed no significant linear association between age and effect size (*β* = −0.046, 95% CI: −0.122 to 0.029, *p* = 0.193). This model explained only limited between-group variance (Adj *R*^2^ = 12.50%), with substantial residual heterogeneity (*I*^2^_res = 71.36%). This indicates that age alone cannot fully account for the variability in MoCA improvement effects.

In contrast, meta-regression analysis for total intervention duration revealed a significant positive correlation with effect size (*β* = 0.00033, 95% CI: 0.00009 to 0.00058, *p* = 0.013). This model explained a substantial proportion of variance (Adj *R*^2^ = 70.08%), with reduced residual heterogeneity (*I*^2^_res = 45.55%). This indicates that longer intervention duration correlates with greater MoCA score improvement, and that dose variation partially accounts for inter-study heterogeneity.

**Figure 8:**
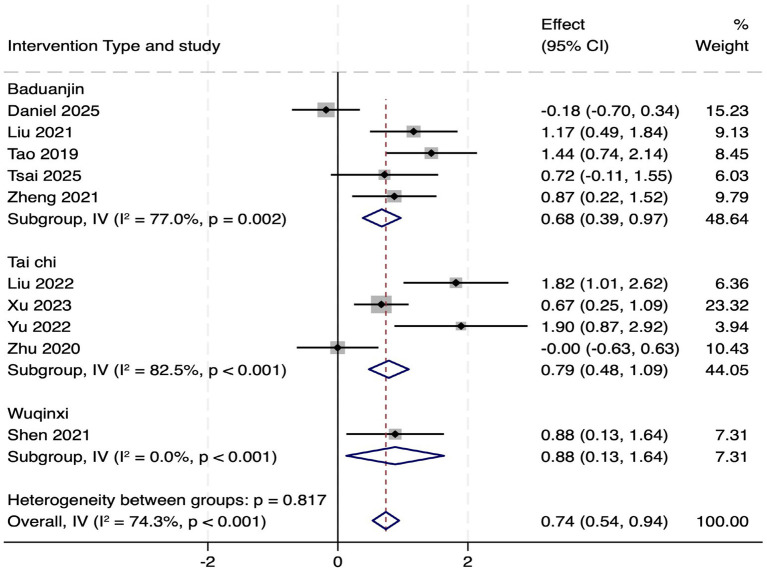
Subgroup analysis forest map of MOCA scale by intervention type.

Subgroup analysis by TMBE type revealed that all three types significantly improved MoCA scores (Figure 8), with no statistically significant differences between subgroups (*p* = 0.817). However, substantial heterogeneity persisted within the Baduanjin (*I*^2^ = 77.0%) and Tai Chi (*I*^2^ = 82.5%) subgroups, suggesting factors beyond exercise type contribute to variability. The Wuqinxi subgroup included only one study, limiting interpretability.

### 3.5 Secondary outcomes

#### 3.5.1 Executive function

This study systematically measured executive function using five indicators: Digit Span Backward, the Chinese and Original versions of TMT-B, the TMT B-A index, and SCWT. We combined results from different versions of the TMT-B and Stroop tests, including Chinese and original version, the premise that they measure the same higher-order cognitive constructs, such as cognitive flexibility and conflict inhibition. Despite potential differences in cultural or procedural details, we consider their conceptual and functional equivalence sufficient to support the combined analysis.

For the Digit Span Backward test, which reflects working memory, results indicated a significant improvement post-intervention (MD = 0.24, 95% CI: 0.05 to 0.44, *p* = 0.013), with low heterogeneity (*I*^2^ = 27.0%, *p* = 0.232) (Figure 8). For indicators assessing cognitive flexibility and task switching, the Chinese TMT-B score improved significantly (MD = −0.34, 95% CI: −0.51 to −0.17, *p* = 0.000), with no heterogeneity (*I*^2^ = 0%, *p* = 0.840). The original TMT-B (TMT-B Original) results showed high heterogeneity (*I*^2^ = 97.0%, *p* < 0.001), but after sensitivity analysis excluding Liu et al. (46), the effect size stabilized and showed significant improvement (MD = −1.18, 95% CI: −1.70 to −0.67, *p* = 0.000), with heterogeneity completely eliminated (*I*^2^ = 0%, *p* = 0.666).

Sensitivity analysis revealed that heterogeneity disappeared when the study by Liu et al. (46) was excluded. This study targeted individuals with MCI and employed exergaming-based tai chi, which differed significantly from the traditional group instruction models used in other studies in terms of intervention format and cognitive load. This disparity likely represents the primary cause of the observed heterogeneity.

The TMT B-A difference score also showed significant improvement (MD = −0.57, 95% CI: −0.99 to −0.15, *p* = 0.210), with acceptable heterogeneity (*I*^2^ = 30.1%, *p* = 0.232). In the Stroop test, which measures conflict inhibition, the SCWT part showed high overall heterogeneity (*I*^2^ = 91.9%, *p* < 0.001). Stroop test formats include color naming, word reading and scoring methods. After excluding Liu et al. (46), heterogeneity disappeared (*I*^2^ = 0%, *p* = 0.473), and the results became more robust. The Word Reading part (Stroop Test Word) also showed high heterogeneity (*I*^2^ = 94.4%, *p* < 0.001). After sensitivity analysis excluding Liu et al. (46), heterogeneity was resolved (*I*^2^ = 0%, *p* = 0.572), enhancing the stability of the results.

This further demonstrates that unconventional intervention formats, such as exergaming, may be key factors contributing to discrepancies with traditional TMBE research findings.

**Figure 9:**
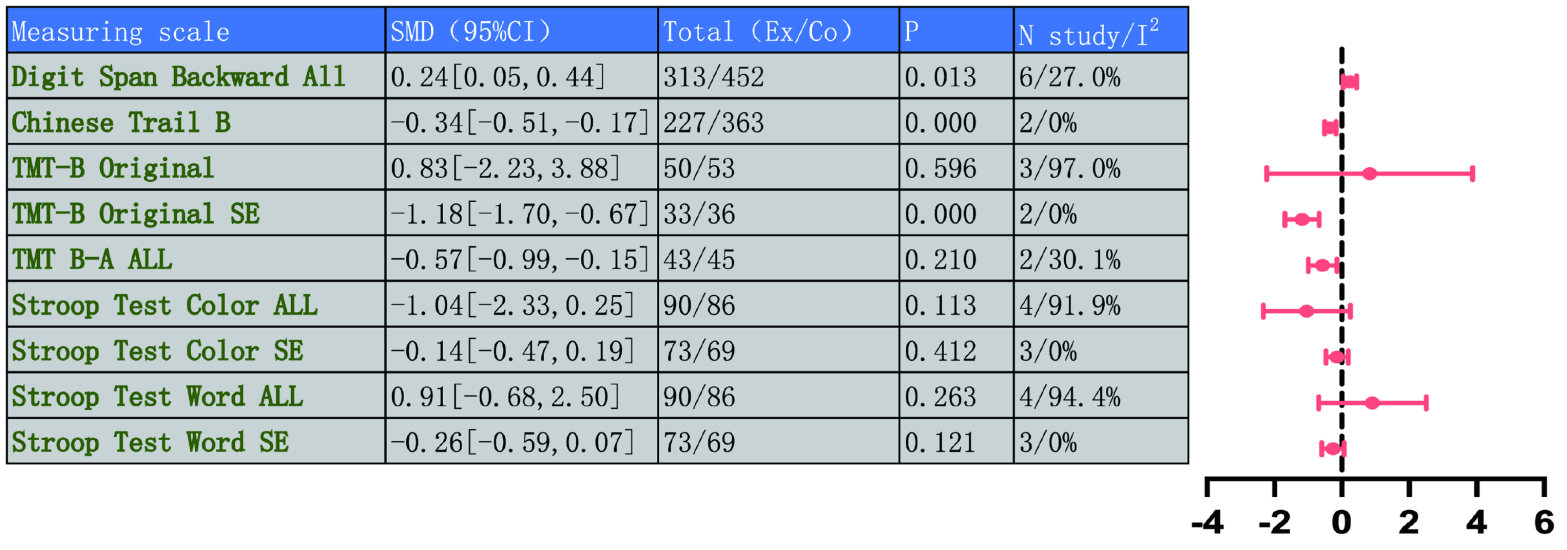
Forest plot collection for executive function.

These results indicate that TMBE can effectively enhance patients’ executive function in working memory, cognitive flexibility, and inhibitory control, demonstrating its effectiveness as a non-pharmacological intervention. Although some original results had heterogeneity, the effect sizes stabilized after sensitivity analysis, which strengthens the reliability of the study conclusions. Detailed forest plots are showed in Figure 9 and Supplementary Figures S2A–L.

#### 3.5.2 Language ability

This study used Verbal Fluency Tests to assess the intervention effect of TMBE on language function. Four studies were included, involving 264 participants in the Ex and 397 in the Co, which demonstrated obvious improvement in verbal fluency performance post-intervention (MD = 0.36, 95% CI: 0.14 to 0.57, *p* = 0.001), with minimal between-study heterogeneity (*I*^2^ = 30.4%, *p* = 0.230), indicating robust results. It should be noted that this analysis is based on only four studies. Although the heterogeneity is low, the stability of the results still requires further studies to validate in populations with prodromal cognitive decline. This finding suggests that TMBE demonstrates benefits in promoting language abilities among patients with NDDs. See Figure 9 and Supplementary Figure S3A for detailed results.

#### 3.5.3 Memory function

The AVLT was used to assess multiple dimensions of memory function, covering IR and LDR. Three studies involving 225 participants were included to analyze immediate recall performance, and another seven studies covering 708 participants focused on LDR to comprehensively reflect changes in memory function.

Overall analysis of immediate recall showed no significant improvement between control and intervention groups (MD = −0.04, 95% CI: −0.54 to 0.47, *p* = 0.890). The analysis revealed substantial heterogeneity (*I*^2^ = 71.8%, *p* = 0.029) (Figure 9). This inconsistency may stem from differences in the sensitivity of specific tests used to assess immediate recall, such as different versions of the AVLT, or from population variations in immediate responses to interventions.

Given that this analysis included only three studies and exhibited significant heterogeneity, there is currently insufficient evidence to demonstrate a significant effect of TMBE on immediate recall. This finding should be regarded as preliminary.

We undertook sensitivity analyses to pinpoint the origins of the observed heterogeneity. After excluding Xu et al. (49), heterogeneity abated significantly in the remaining study subset (*I*^2^ = 30.8%, *p* = 0.229), but the pooled effect size remained statistically non-significant (MD = 0.19, 95% CI: −0.22 to 0.60, *p* = 0.365), suggesting that TMBE may not have significantly improved immediate recall function among people with NDDs or prodromal cognitive decline.

**Figure 10:**
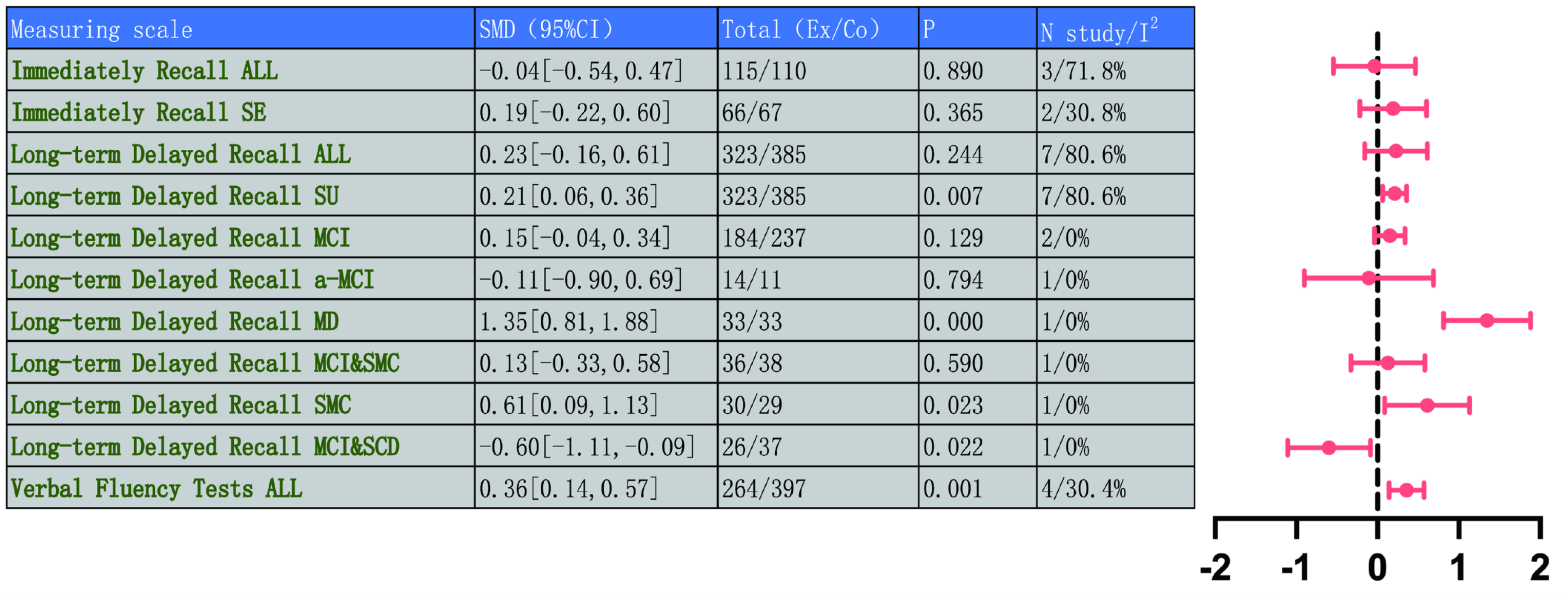
Comprehensive forest plots of memory function and language ability.

For long-term delayed recall, the overall analysis of 7 studies (*n* = 708 participants) indicated no significant improvement (MD = 0.23, 95% CI: −0.16 to 0.61, *p* = 0.244), considerable heterogeneity (*I*^2^ = 80.6%, *p* < 0.001) was observed. However, subgroup analysis based on disease type revealed a clear pattern, a significant portion of the heterogeneity could be explained by disease stage. Analysis focusing on specific populations found the most significant benefit in patients with Mild Dementia (MD = 1.35, 95% CI: 0.81 to 1.88, *p* = 0.000). Participants with Subjective Memory Complaint (SMC) were significantly improved (MD = 0.61, 95% CI: 0.09 to 1.13, *p* = 0.023). In contrast, a clear adverse effect was observed within the MCI&SCD subgroup (MD = −0.60, 95% CI: −1.11 to −0.09, *p* = 0.022). No significant effects were found in analyses limited to MCI or a-MCI populations. The result from a single study by Grzenda et al. (50) is the primary cause of overall heterogeneity and non-significant results. This contradictory finding highlights the potential for significant differences in TMBE responses across populations with varying pre-treatment statuses, warranting attention in future research. In summary, the role of TMBE in enhancing long-term delayed recall is not uniform but varies substantially across different populations with cognitive impairment. Detailed results are shown in Figure 10 and Supplementary Figures S4A–E.

#### 3.5.4 Attention function

Four indicators were used to assess basic attention function: Digit Span Forward, the Chinese and Original versions of TMT-A, and Visual Span Forward.

It should be noted that this study conducted a pooled meta-analysis of the Chinese version and the original TMT-A. This decision was based on the following premises, these versions measure the same underlying cognitive structures, namely that basic visual attention and processing speed, and share the same unit of measurement.

Three independent studies (total 612 participants, intervention/control: 237/375) used the Digit Span Forward test to assess auditory attention capacity. A random-effects model meta-analysis suggested an increasing trend in scores for the intervention group over control, and yet the difference lacked statistical support (MD = 0.36, 95% CI: 0.05 to 0.67, *p* = 0.021). This analysis had moderate heterogeneity (*I*^2^ = 59.8%, *p* = 0.083). Further sensitivity analysis excluding the source of heterogeneity (47) involved two studies (total 590 participants, intervention/control: 227/363) showing high consistency (*I*^2^ = 0%, *p* = 0.763) and indicated that the intervention significantly improved Digit Span Forward performance (MD = 0.27, 95% CI: 0.10 to 0.43, *p* = 0.002), finding a clear and significant positive effect under homogeneous conditions.

**Figure 11:**
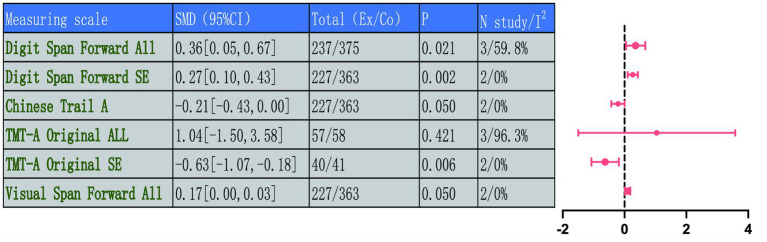
Comprehensive forest plots of attention function.

Furthermore, two studies used the Chinese TMT-A to assess visual attention efficiency, involving the same 590 participants (227/363), with low heterogeneity (*I*^2^ = 0%, *p* = 0.441). Analysis by a random-effects model revealed that completion time of TC group was slightly better than the Co, with a difference that approached statistical significance (MD = −0.21, 95% CI: −0.43 to 0.00, *p* = 0.050). On the other hand, three studies used the original TMT-A (TMT-A Original) for assessment, involving 115 participants (57/58), with results showing high heterogeneity (*I*^2^ = 96.3%, *p* < 0.001). The pooled effect size was no statistical significance (MD = 1.04, 95% CI: −1.50 to 3.58, *p* = 0.421). This high degree of heterogeneity likely stems from significant differences in research methodology, particularly the culturally adapted versions of the TMT-A test itself and variations in baseline characteristics among study populations. After sensitivity analysis excluding Liu et al. (46), two homogeneous studies (*I*^2^ = 0%) indicated that the TMBE intervention significantly shortened TMT-A completion time (MD = −0.63, 95% CI: −1.07 to −0.18, *p* = 0.006). This finding indicates that, after excluding key sources of heterogeneity, the beneficial effect of TMBE on visual attention processing speed is clearly and consistently demonstrated across studies employing more methodologically consistent approaches. Another two RCTs assessed visual information retention using the Visual Span Forward test, involving the same sample size (227/363), with low heterogeneity (*I*^2^ = 0%, *p* = 0.514). Results revealed a slight improvement compared to experimental group (MD = 0.17, 95% CI: 0.00 to 0.34, *p* = 0.050). In summary, traditional mind–body exercise showed positive improving trends for both auditory and visual channel information reception breadth and visual information processing speed. Although some results were influenced by heterogeneity and differences in measurement tools, the stability of effects after sensitivity analysis enhanced the reliability of the results. Detailed results are shown in Figure 11 and related Supplementary Figures S4A–H.

#### 3.5.5 Publication bias

**Figure 12:**
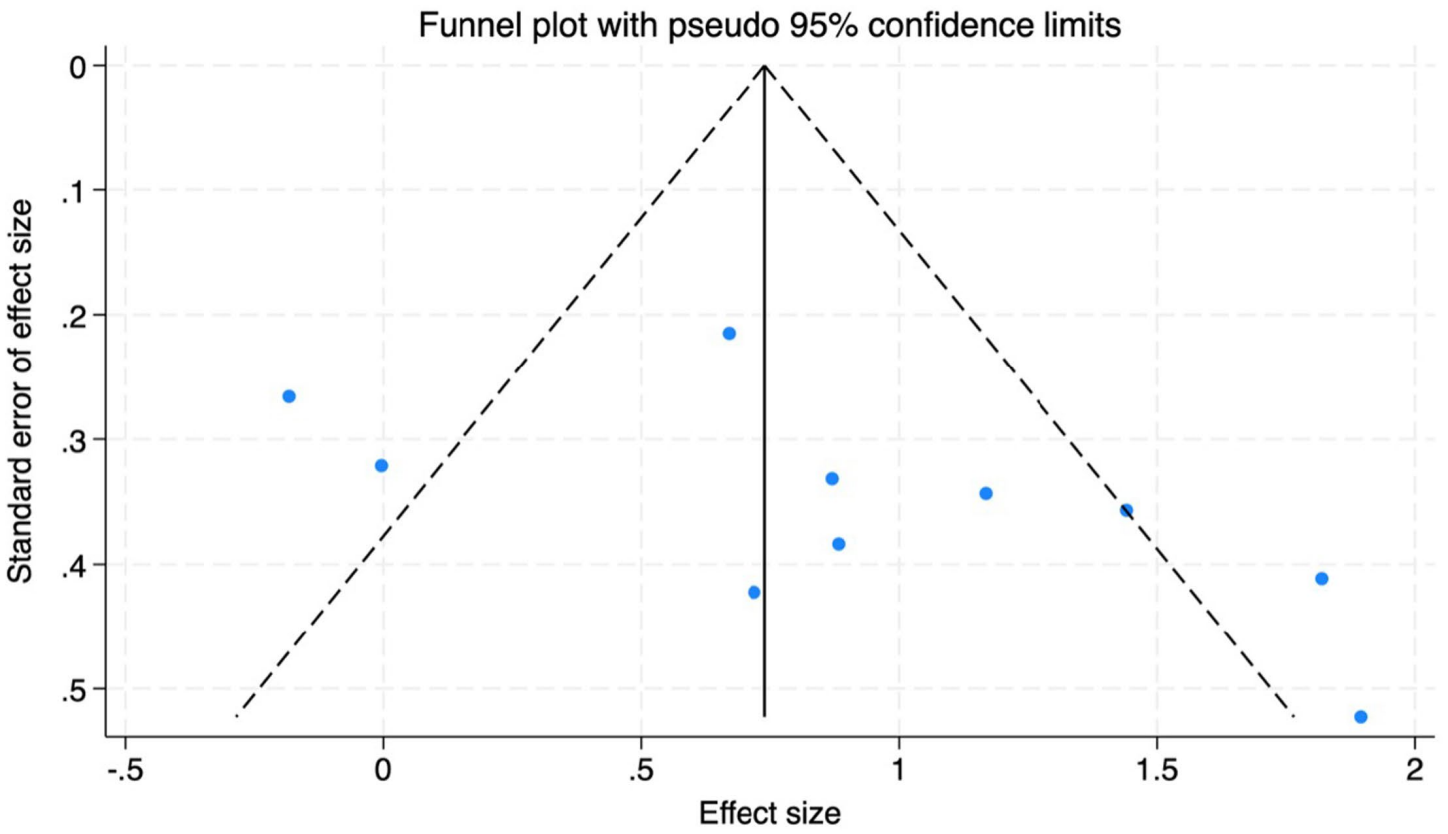
Funnel plot to detect the publication bias of included studies.

**Figure 13:**
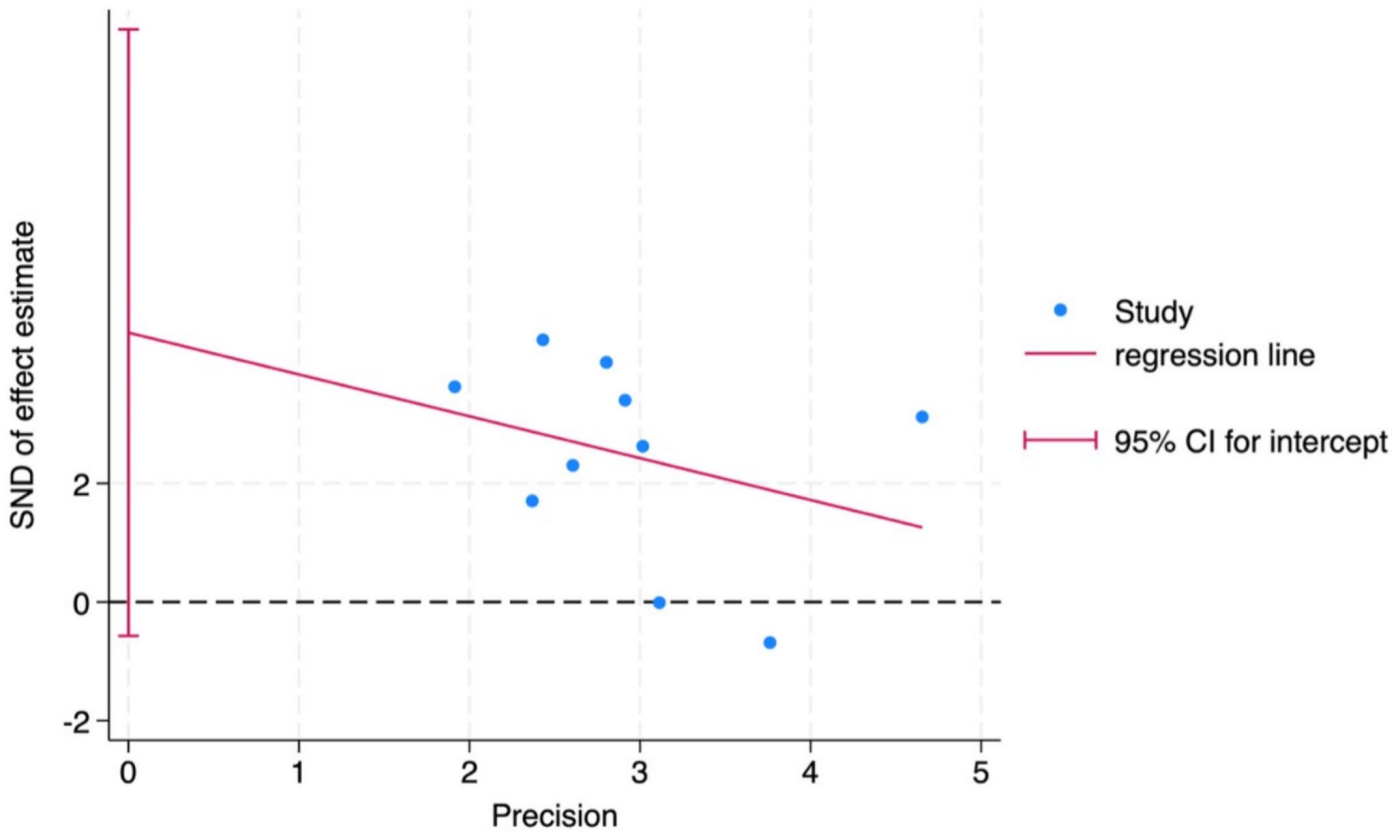
Egger’s linear regression test for publication bias.

To assess potential publication bias, we conducted both visual and quantitative analyses (Figure 12). A funnel plot using MoCA as an indicator showed that the distributional pattern of the included studies on both sides of the pooled effect size, due to the limited proportion of sites that fall outside the range of expected confidence intervals, suggesting the current results do not support the conclusion that there is significant publication bias in this area. Further Egger’s linear regression test results (Figure 13) showed that the confidence interval for the regression line intercept included zero, indicating no significant small-study effects (*t* = 2.05, *p* = 0.075). Combining these results, the results of this meta-analysis show robustness to publication bias. However, it should be noted that when the number of studies included in a given outcome measure is small, as demonstrated in some analyses within this study, the statistical power of these methods to detect publication bias may be insufficient.

## 4 Discussion

### 4.1 Clinical implications

This meta-analysis confirms that TMBE produces significant benefits across multiple core cognitive domains, including executive function, language ability, memory, and attention in patients with NDDs and those in the prodromal stage of cognitive decline.

These findings provide crucial evidence to inform clinical decision-making for this population and establishes a practical foundation for promoting non-pharmacological intervention strategies. Impaired global cognitive function is a core symptom of NDDs and age-related cognitive decline, profoundly compromising the living quality and ability for independent living in affected individuals. In clinical practice, selecting interventions that effectively and consistently improve cognitive function for this population remains an ongoing challenge. The view is supported by the solid evidence from our study, demonstrating that TMBE making global cognitive function elevated, which aligns with findings from previous systematic reviews (23, 24, 53).

The efficacy of TMBE may stem from its multi-component integrated nature. It combines moderate-intensity physical activity, cognitive focus, breathing regulation, and meditation practice, synergistically supporting cognitive health through both physiological and psychological pathways (54, 55). Its gentle nature also ensures good compliance among older adults and those with cognitive impairment (25).

Mechanistically, this integrated intervention may exert effects through multiple pathways, including enhancing cerebral blood flow, regulating neurotrophic factors, strengthening neural connections, and reducing neuroinflammation and stress responses.

When interpreting the findings of this study, it is crucial to consider their clinical significance. Although TMBE produced statistically significant improvements of 0.87 points on the MoCA and 0.65 points on the MMSE, these magnitude effects may be near or below the threshold for minimal clinically important differences, which are typically considered to be one to two points for the MoCA, and two to three points for the MMSE. This suggests that TMBE exerts a positive directional effect on group cognitive function, but its perceptible benefits for individual patients may be subtle. Achieving unequivocal clinical significance may require longer-term, more intensive interventions.

Based on current evidence, therefore, TMBE can be recommended as a clinical cognitive intervention strategy suitable for delaying neurodegenerative cognitive decline.

### 4.2 Interpretation of results by cognitive domain

This study found that TMBE significantly improved multiple core components of executive function, including working memory, cognitive flexibility, and conflict inhibition. This finding aligns with the consensus that executive function represents the cognitive domain most amenable to training effects (56). Such broad improvements are likely directly driven by the multitasking nature of TMBE, practitioners must simultaneously coordinate complex movement sequences while maintaining cognitive focus and regulating respiration. This process continuously challenges and activates the prefrontal cortex—the critical neural substrate underpinning executive function (57–59).

This study also revealed important negative and heterogeneous findings. TMBE showed no significant effect on immediate recall, potentially because this cognitive dimension relies more on medial temporal lobe structural integrity (60), and is less sensitive to interventions primarily challenging prefrontal cortex and executive function. More notably, the negative effect observed on long-delayed recall in the MCI&SCD subgroup (50), strongly suggests significant biological or psychological heterogeneity within the preclinical cognitive decline population. For some individuals, complex mind–body exercises may introduce cognitive load rather than provide support during early intervention phases. These findings caution that the cognitive benefits of TMBE are not universal, underscoring the urgent need for future research to identify optimal responders versus potential non-responders.

Unlike its effects on executive functions, TMBE demonstrates marked dimensional specificity and population heterogeneity in enhancing memory functions. A key finding is that TMBE exerts no significant impact on immediate recall. This may stem from the greater dependence of immediate recall on structural integrity within the medial temporal lobe, such as the hippocampus (61–63). Interventions targeting the prefrontal cortex show lower sensitivity to major challenges (59). More importantly, the long-delayed recall data present a complex pattern, despite observing significant and consistent benefits in individuals with MCI and SMC, a distinct negative effect emerged in the subgroup with MCI&SCD. This heterogeneous finding strongly suggests that distinct populations at subtle stages of cognitive decline may exhibit fundamentally different physiological and psychological responses to TMBE. For some MCI&SCD individuals, the complex Mind–Body practice may have exceeded their cognitive resources during the early intervention phase, introducing cognitive load rather than providing support. Therefore, treating prodromal cognitive impairment as a homogeneous group is inappropriate; this finding underscores the importance of distinguishing responders from non-responders in future studies and exploring the underlying neural mechanisms. Notably, TMBE demonstrated significant memory improvement in patients with mild dementia, whereas its effects were relatively limited in individuals with mild cognitive impairment. This differential response may stem from the relatively intact memory circuits and higher baseline memory performance of the latter group. This aligns with the view proposed by Liu et al. (64) that individuals with more pronounced cognitive impairment can achieve greater cognitive benefits. Furthermore, extending the intervention cycle may enhance the memory-improving effects of TMBE.

This analysis demonstrates that TMBE consistently enhances language abilities, such as verbal fluency (7). Given that language production heavily relies on executive control for lexical retrieval and memory systems (65, 66). The benefits of TMBE on language function are likely not mediated directly, but rather indirectly through its effects on improving executive function and episodic memory.

The effects of TMBE on attention are the most complex, with its efficacy assessment highly dependent on the tools employed. One of the most significant findings is that TMBE demonstrates a consistent improvement in auditory attention, as indexed by forward digit span (Figure 11), while its benefits for visual processing speed are inconsistent and modest by TMT-A. This discrepancy may reflect the differential effects of TMBE components, exercises involving verbal instructions and rhythmic control appear to enhance auditory processing more strongly (67), while its impact on fundamental visual search efficiency is limited. Thus, TMBE may not constitute a broad-spectrum attention intervention (68), and clinicians should consider its application based on the specific attention deficit dimension of patients, such as auditory or visual.

### 4.3 Study limitations

A primary limitation is the substantial heterogeneity observed across multiple analyses. Although anticipated and addressed through random-effects models and subgroup analyses, its presence cautions against overgeneralizing conclusions. This heterogeneity stems from the study design, which intentionally included a wide-ranging patient population, ranging from SCD to mild dementia, diverse TMBE intervention protocols varying in type, dosage, and guidance quality, and the versions and cultural adaptations of cognitive assessment tools employed. Although our sensitivity analyses successfully identified specific sources such as exergaming-based interventions, residual heterogeneity indicates that unmeasured effect-modifying factors play a role, such as genetic background, specific pathology, and sociocultural context.

Another issue is the lack of detailed raw data, which prevents us from conducting in-depth analyses of specific factors influencing treatment efficacy. For example, the absence of information on patient comorbidities, gender, or genetic background limits our ability to explore personalized treatment approaches. It is important to note that the findings of this study may be more applicable to East Asian populations. Most research has been conducted in this region, and the effectiveness of different mind–body exercise modalities may be influenced by cultural contexts. Furthermore, blinding patients and coaches in such exercise studies is challenging, potentially introducing bias into the findings.

To address these limitations, future research should conduct larger-scale, longer-term clinical trials. We recommend directly comparing the efficacy of different exercise modalities to provide patients with more precise exercise regimens. Additionally, we explored the effects of age, intervention duration, and exercise type on heterogeneity through meta-regression and subgroup analysis, other potential moderating factors could not be fully analyzed due to inconsistencies across studies. Moreover, the limited number of yoga intervention studies and the lack of standardized assessment tools constrained our ability to conduct independent subgroup analyses for this intervention type.

## 5 Conclusion

This meta-analysis indicates that TMBE represent a promising non-pharmacological intervention strategy for cognitive health in individuals with NDDs or those at the prodromal stage of cognitive decline. It demonstrates significant improvement effects on overall cognitive function, executive function, language abilities, and long-term memory in specific subgroups. However, its efficacy varies considerably across different cognitive domains and patient populations, and it is not effective for all measured cognitive dimensions. Future research should focus on optimizing intervention protocols, elucidating underlying mechanisms, and employing personalized medicine approaches to identify populations most likely to benefit. To achieve these objectives, future research should prioritize standardized reporting of intervention parameters and participant characteristics to support more refined meta-regression and moderator analyses. Concurrently, in-depth exploration of interactions between intervention type, dose, and individual characteristics is essential for developing personalized TMBE recommendation protocols.

## Data Availability

The original contributions presented in the study are included in the article/Supplementary material, further inquiries can be directed to the corresponding author.

## Glossary

AD: Alzheimer’s Disease
ALL: Overall meta-analysis
a-MCI: Amnestic Mild Cognitive Impairment
AVLT: Auditory Verbal Learning Test
CDR: Clinical Dementia Rating
CDR-SB: Clinical Dementia Rating-Sum of Boxes
Co: Control group
Ex: Experimental group
HD: Huntington’s Disease
IR: Immediate Recall
LDR: Long-term Delayed Recall
Long-term Delayed Recall MD: Long-term Delayed Recall Mild Dementia
MCI: Mild Cognitive Impairment
MCI&SCD: Mild Cognitive Impairment and Subjective Cognitive Decline
MCI&SMC: Mild Cognitive Impairment and Subjective memory complaints
MMSE: Mini-Mental State Examination
MMSE MD: Mini-Mental State Examination Mild Dementia
MoCA: Montreal Cognitive Assessment
MQ: Memory Quotient
PD: Parkinson’s Disease
SCD: Subjective Cognitive Decline
SCWT: Stroop Color and Word Test
SE: Sensitivity Analysis
SMC: Subjective Memory Complaints
SU: Sub-group Analysis
TMT A: Trail Making Test A
TMT B: Trail Making Test B
TMT B-A: Trail Making Test B-A
WMS-CR: Wechsler Memory Scale – Chinese Revised
