# Supplementary material for: The effects of traditional mind-body exercises on cognitive function in neurodegenerative diseases or prodromal cognitive decline: a meta-analysis
Source: Front Public Health. 2026 Feb 11;14:1735606. doi: 10.3389/fpubh.2026.1735606 (PMC12932201; doi:10.3389/fpubh.2026.1735606)
Supplement: Supplementary file 1 [file Table_1.pdf]

Supplementary Material

Table S1. Literature Search Strategy

**Pubmed**	Number of studies retrieved: 156<br><br>1 ((((((((((Tai-ji[Title/Abstract]) OR (Tai Chi[Title/Abstract])) OR (Chi, Tai[Title/Abstract])) OR (Tai Chi Chuan[Title/Abstract])) OR (Taiji[Title/Abstract])) OR (Taijiquan[Title/Abstract])) OR (T'ai Chi[Title/Abstract])) OR (Tai Ji Quan[Title/Abstract])) OR (Ji Quan, Tai[Title/Abstract])) OR (Quan, Tai Ji[Title/Abstract])<br><br>2 Wuqinxi[Title/Abstract] OR five animal frolics[Title/Abstract] OR wu qin xi[Title/Abstract]<br><br>3 ((((Baduanjin[Title/Abstract]) OR (Ba Duan Jin[Title/Abstract])) OR (Ba-Duan-Jin[Title/Abstrac])) OR (ba duan jin[Title/Abstract])) OR (Eight Section Brocade[Title/Abstract])<br><br>4 yijinjing[Title/Abstract]<br><br>5 liuzijue[Title/Abstract]<br><br>6 (Qigong[Title/Abstract]) OR (Ch'i Kung[Title/Abstract])) OR (qi gong[Title/Abstract])<br><br>7 yoga[Title/Abstract]<br><br>8 ((((mind-body exercise*[Title/Abstract]) OR (traditional exercise*[Title/Abstract])) OR (mind-bod y therap*[Title/Abstract])) OR (meditative movement*[Title/Abstract])) OR (chinese exercise*[Titl e/Abstract])<br><br>9 (((((((#1) OR (#2)) OR (#3)) OR (#4)) OR (#5)) OR (#6)) OR (#7)) OR (#8)<br><br>10 (((((((((((("Neurodegenerative Diseases"[Mesh])) OR "Alzheimer Disease"[Mesh]) OR "Parkinson Disease"[Mesh]) OR "Dementia"[Mesh]) OR "Cognitive Dysfunction"[Mesh]) OR (neurodegenerat*[Title/Abstract])) OR (alzheimer*[Title/Abstract])) OR (parkinson*[Title/Abstract])) OR (dementi

		a[Title/Abstract])) OR (lewy bod*[Title/Abstract])) OR (mild cognitive impair*[Title/Abstract])) OR (MCI[Title/Abstract])) OR (cognitive decline[Title/Abstract])
	11	(#9) AND (#10)
	12	((((((((((("Cognition"[Mesh]) OR "Executive Function"[Mesh]) OR "Memory"[Mesh]) OR "Attention"[Mesh]) OR "Neuropsychological Tests"[Mesh]) OR (cognit*[Title/Abstract])) OR (executive function*[Title/Abstract])) OR (memory[Title/Abstract])) OR (attention[Title/Abstract])) OR (neuropsycholog*[Title/Abstract])) OR (MMSE[Title/Abstract])) OR (MoCA[Title/Abstract])) OR (cognitive test*[Title/Abstract])
	13	(#11) AND (#12)
	14	randomized controlled trial*[Title/Abstract]
	15	(#13) AND (#14)
**Web of Science**		Number of studies retrieved: 416<br><br>1: (TI=(Tai-ji OR Tai Chi OR Chi, Tai OR Tai Chi Chuan OR Taiji OR Taijiquan OR T'ai Chi OR Tai Ji Quan OR Ji Quan, Tai OR Quan, Tai Ji OR Wuqinxi OR five animal frolics OR wu qin xi OR Baduanjin OR Ba Duan Jin OR Ba-Duan-Jin OR ba duan jin OR Eight Section Brocade OR yijinjing OR liuzijue OR Qigong OR Ch'i Kung OR qi gong OR yoga OR mind-body exercise* OR traditional exercise* OR mind-body therap* OR meditative movement* OR chinese exercise*)) OR AB=(Tai-ji OR Tai Chi OR Chi, Tai OR Tai Chi Chuan OR Taiji OR Taijiquan OR T'ai Chi OR Tai Ji Quan OR Ji Quan, Tai OR Quan, Tai Ji OR Wuqinxi OR five animal frolics OR wu qin xi OR Baduanjin OR Ba Duan Jin OR Ba-Duan-Jin OR ba duan jin OR Eight Section Brocade OR yijinjing OR liuzijue OR Qigong OR Ch'i Kung OR qi gong OR yoga OR mind-body exercise* OR traditional exercise* OR mind-body therap* OR meditative movement* OR chinese exercise*)<br><br>2: (TI=(neurodegenerat* OR alzheimer* OR parkinson* OR dementia OR lewy bod* OR mild cognitive impair* OR MCI OR cognitive decline)) OR AB=(neurodegenerat* OR alzheimer* OR parkinson* OR dementia OR lewy bod* OR mild cognitive impair* OR MCI OR cognitive decline)

	3: (TI=(cognit* OR executive function* OR memory OR attention OR neuropsycholog* OR MMSE OR MoCA OR cognitive test*)) OR AB=(cognit* OR executive function* OR memory OR attention OR neuropsycholog* OR MMSE OR MoCA OR cognitive test*)<br><br>4: (TI=(randomized controlled trial*)) OR AB=(randomized controlled trial*)<br><br>5：#1 AND #2 AND #3 AND #4
**Embase**	Number of studies retrieved: 527<br><br>#1 'tai ji'/exp<br><br>#2 'tai chi'/exp<br><br>#3 'yoga'/exp<br><br>#4 'qigong'/exp<br><br>#5 'breathing exercise'/exp<br><br>#6 tai ji':ti,ab OR 'taiji':ti,ab OR 'tai chi':ti,ab OR 'taichi':ti,ab OR 'tai chi chuan':ti,ab OR 'qigong':ti,ab OR 'chi kung':ti,ab OR 'qi gong':ti,ab OR 'baduanjin':ti,ab OR 'ba duan jin':ti,ab OR 'wuqinxi':ti,ab OR 'wu qin xi':ti,ab OR 'yoga':ti,ab OR 'mind-body exercise*':ti,ab OR 'traditional exercise*':ti,ab OR 'mind-body therap*':ti,ab OR 'meditative movement*':ti,ab OR 'chinese exercise*':ti,ab

#7	#1 OR #2 OR #3 OR #4 OR #5 OR #6
#8	'neurodegenerative disease'/exp
#9	'alzheimer disease'/exp
#10	'parkinson disease'/exp
#11	'dementia'/exp
#12	'cognitive defect'/exp
#13	'neurodegenerat*':ti,ab OR 'alzheimer*':ti,ab OR 'parkinson*':ti,ab OR 'dementia':ti,ab OR 'lewy bod*':ti,ab OR 'mild cognitive impair*':ti,ab OR 'mci':ti,ab OR 'cognitive decline':ti,ab
#14	#8 OR #9 OR #10 OR #11 OR #12 OR #13
#15	'cognition'/exp
#16	'cognitive defect'/exp
#15	'executive function'/exp
#16	'memory'/exp
#17	'attention'/exp

	#18 'neuropsychological test'/exp<br><br>#19 'cognit*':ti,ab OR 'executive function*':ti,ab OR 'memory':ti,ab OR 'attention':ti,ab OR 'neuropsycholog*':ti,ab OR 'mmse':ti,ab OR 'moca':ti,ab OR 'cognitive test*':ti,ab<br><br>#20 (cognit* or executive function* or memory or attention or neuropsycholog* or MMSE or MoCA or cognitive test*):ti,ab,kw<br><br>#21 #15 OR #16 OR #17 OR #18 OR #19 OR #20 OR<br><br>#22 #7 AND #14 AND #22<br><br>#23 'randomized controlled trial'/exp OR 'randomized controlled trial'<br><br>#24 #22 AND #23
**Cochrane**	Number of studies retrieved:323<br><br>#1 MeSH descriptor: [Tai Ji] explode all trees<br><br>#2 MeSH descriptor: [Yoga] explode all trees<br><br>#3 MeSH descriptor: [Qigong] explode all trees<br><br>#4 (taiji or tai ji or tai chi or taichi or tai chi quan) : ti,ab,kw OR (qigong or chi kung or qi gong):ti,ab,kw OR (baduanjin or ba duan jin):ti,ab,kw OR (wuqinxi or wu qin xi):ti,ab,kw OR (yoga):ti,ab,kw

#5	(mind-body exercise* or traditional exercise* or mind-body therap* or meditative movement* or chinese exercise*):ti,ab,kw
#6	#1 OR #2 OR #3 OR #4 OR #5
#7	MeSH descriptor: [Neurodegenerative Diseases] explode all trees
#8	MeSH descriptor: [Alzheimer Disease] explode all trees
#9	MeSH descriptor: [Parkinson Disease] explode all trees
#10	MeSH descriptor: [Dementia] explode all trees
#11	(neurodegenerat*):ti,ab,kw OR (alzheimer*):ti,ab,kw OR (parkinson*):ti,ab,kw OR (dementia):ti,ab,kw OR (lewy bod* or mild cognitive impair* or MCI or cognitive declin):ti,ab,kw
#12	#7 OR #8 OR #9 OR #10 OR #11
#13	#6 AND #12
#14	MeSH descriptor: [Cognition] explode all trees
#15	MeSH descriptor: [Cognitive Dysfunction] explode all trees
#16	MeSH descriptor: [Executive Function] explode all trees
#17	MeSH descriptor: [Memory] explode all trees
#18	MeSH descriptor: [Attention] explode all trees
#19	MeSH descriptor: [Neuropsychological Tests] explode all trees
#20	(cognit* or executive function* or memory or attention or neuropsycholog* or MMSE or MoCA or cognitive test*):ti,ab,kw

#21	#14 OR #15 OR #16 OR #17 OR #18 OR#19 OR #20
#22	#13 AND #21
#23	randomized controlled trial*
#24	#22 AND #23
