# Supplementary material for: The effects of traditional mind-body exercises on cognitive function in neurodegenerative diseases or prodromal cognitive decline: a meta-analysis
Source: Front Public Health. 2026 Feb 11;14:1735606. doi: 10.3389/fpubh.2026.1735606 (PMC12932201; doi:10.3389/fpubh.2026.1735606)
Supplement: Supplementary file 2 [file Supplementary_file_1.docx]

Supplementary Material

# 1 Supplementary Figure S1. Bubble plots of meta-regression analyses

(A) Meta-regression of mean participant age on Moca scores

(B) Meta-regression of total intervention duration on Moca scores

# 2 Supplementary Figure S2. Executive function

(**A**) Forest map of digit span backward test

(B）Forest map of chinese trail B test

(C) Forest map of TMT-B original test

(D) Sensitivity analysis of TMT-B original test

(E) Forest map after sensitivity analysis of TMT-B original test

(F) Forest map of TMT B-A test

(G) Forest map of stroop test color

(H) Sensitivity analysis of stroop test color

(I) Forest map after sensitivity analysis of stroop test color

(J) Forest map of stroop test word

(K) Sensitivity analysis of stroop test word

(L) Forest map after sensitivity analysis of stroop test word

**Supplementary Figure S3.** Language ability

(A) Forest map of categorical verbal fluency tests

**Supplementary Figure S4.** Memory function

(A) Forest map of immediately recall test

(B) Sensitivity analysis of immediately recall test

(C) Forest map after sensitivity analysis of immediately recall test

(D) Forest map of long-term delayed recall test

(E) Subgroup analysis forest map of long-term delayed recall test

**Supplementary Figure S4.** Attention function

(A) Forest map of digit span forward test

(B) Sensitivity analysis of digit span forward test

(C) Forest map after sensitivity analysis of digit span forward test

(D) Forest map of chinese trail A test

(E) Forest map of TMT-A Original test

(F) Sensitivity analysis of TMT-A Original test

(G) Forest map after sensitivity analysis of TMT-A Original test

(H) Forest map of visual span forward test
